# PPM1D is directly degraded by proteasomes in a ubiquitination-independent manner through its carboxyl-terminal region

**DOI:** 10.1186/s12929-025-01185-z

**Published:** 2025-09-11

**Authors:** Masaki Takahashi, Takeshi Kondo, Shogo Kimura, Akira Nakazono, Shusei Yoshida, Takeshi Wada, Masashi Watanabe, Shigetsugu Hatakeyama

**Affiliations:** 1 Department of Biochemistry, Faculty of Medicine and Graduate School of Medicine, Hokkaido University, Sapporo, Japan; 2 Division of Acute and Critical Care Medicine, Department of Anaesthesiology and Critical Care Medicine, Faculty of Medicine and Graduate School of Medicine, Hokkaido University, Sapporo, Japan; 3 Department of Otolaryngology-Head and Neck Surgery, Faculty of Medicine and Graduate School of Medicine, Hokkaido University, Sapporo, Japan

**Keywords:** PPM1D, Proteasome, UbInPD, Bortezomib, PSMD14, PSME3, GSK2830371

## Abstract

**Background:**

PPM1D (protein phosphatase Mg^2^⁺/Mn^2^⁺ dependent 1D) is a Ser/Thr phosphatase that negatively regulates p53 and functions as an oncogenic driver. Its gene amplification and overexpression are frequently observed in various malignancies and disruption of PPM1D degradation has also been reported as a cause of cancer progression. However, the precise mechanisms regulating PPM1D stability remain to be elucidated.

**Methods:**

PPM1D stability and degradation pathways were examined using cycloheximide chase assays in multiple cell lines. Proteasome and lysosome inhibitors were used to determine the degradation mechanism, while ubiquitination dependency was assessed using TAK-243, an E1 ubiquitin-activating enzyme inhibitor. In vitro degradation assays with purified 20S proteasome were performed to evaluate direct proteasomal degradation. Immunoprecipitation followed by mass spectrometry was performed to identify proteasomal regulators of PPM1D, with their functional roles validated through knockdown experiments. Finally, cell viability assays were conducted to assess the therapeutic potential of combined proteasome and PPM1D inhibition.

**Results:**

Cycloheximide chase assays demonstrated that wild-type PPM1D is a short-lived protein, whereas a C-terminal truncation mutant exhibits increased stability. PPM1D undergoes rapid, ubiquitin-independent proteasomal degradation via its C-terminal 35 amino acid residues. Additionally, the region spanning residues 450–501 is necessary for ubiquitination-mediated suppression of the ubiquitin-independent degradation pathway. We also found that PPM1D is directly degraded by the 20S proteasome, with the regulatory proteasome subunits PSMD14 and PSME3 acting as activators in this process. Proteasome inhibition resulted in PPM1D accumulation, potentially reducing therapeutic efficacy. Combined proteasome and PPM1D inhibition synergistically enhanced the antitumor effect.

**Conclusions:**

The rapid degradation of the cancer driver PPM1D is achieved through direct recognition by the proteasome, and proteasome inhibitors may reduce therapeutic efficacy due to the accumulation of PPM1D. PPM1D may serve as a suitable model substrate for elucidating the mechanism of ubiquitin-independent proteasomal degradation and represents a potential novel therapeutic target for cancer treatment based on proteasome inhibition.

**Supplementary Information:**

The online version contains supplementary material available at 10.1186/s12929-025-01185-z.

## Background

PPM1D (protein phosphatase Mg^2^⁺/Mn^2^⁺ dependent 1D), also known as wild-type p53-induced phosphatase 1, is a Ser/Thr protein phosphatase induced by wild-type p53 in response to cellular stress, such as DNA damage [1]. PPM1D negatively regulates p53 by dephosphorylation, forming a negative feedback loop that prevents excessive p53 activation. Through this mechanism, PPM1D plays a role in cell cycle regulation, promotion of cell proliferation, induction of DNA repair, and inhibition of apoptosis [2]. PPM1D is an oncogene in Tier 1 of the COSMIC (Catalogue of Somatic Mutations in Cancer) cancer gene census [3]. Gene amplification and overexpression of PPM1D have been reported in various malignancies, including breast cancer, lung cancer, and haematological malignancies, contributing to cancer progression and metastasis [2]. Cancer-associated PPM1D mutations often produce C-terminally truncated proteins that lack degradation signals, resulting in increased stability and activity. These mutants constitutively suppress p53 signaling and promote cancer progression in various malignancies [4–6].

The degradation mechanism of PPM1D has been partially elucidated. A study has shown that HIPK2 phosphorylates PPM1D at serine residues 54 and 85, thereby promoting its ubiquitin-dependent proteasomal degradation, although the specific ubiquitin ligase involved remains unidentified [7]. Another study has reported that in HCT116 (p.L450X) and U2OS (p.R458X) cells heterozygously expressing both wild-type PPM1D and a C-terminal truncation mutant, the mutant protein is expressed at levels 10- to 20-fold higher than wild-type PPM1D due to increased protein stability. Furthermore, treatment with proteasome inhibitors led to stabilization of wild-type PPM1D within cells, suggesting that its C-terminal domain serves as a key target for proteasome-mediated degradation [4]. Despite the fact that disruption of the PPM1D degradation mechanism strongly promotes cancer progression, the precise mechanism underlying the proteasomal degradation of PPM1D remains incompletely understood.

While the majority of eukaryotic proteins undergo proteasomal degradation through ubiquitination, a subset has been demonstrated to be subject to ubiquitin-independent proteasomal degradation (UbInPD). Previous studies have proposed several proteins, including ODC1, p53, p73, c-Myc [8], and p21, as substrates of UbInPD [9–11]. Recent research has significantly expanded our understanding of this process, identifying a considerable number of additional substrates [12]. However, the complete landscape of ubiquitin-independent protein degradation remains to be fully elucidated. This is particularly evident given the estimation that up to 20% of intracellular proteins may undergo degradation independently of ubiquitination [13]. Over the years, significant efforts have been dedicated to elucidating the molecular mechanisms by which UbInPD substrates are targeted to the proteasome. Specific sequences at the C-terminus, intrinsically disordered regions within the substrate, and defined sequence motifs containing hydrophobic residues have been shown to enhance proteasomal recognition and degradation [12, 14, 15]. However, due to the relatively limited number of identified substrates, it remains unclear whether the few known UbInPD substrates are representative of all classes of such substrates. Furthermore, for many of these proteins, the evidence supporting ubiquitin-independent degradation has been primarily obtained through in vitro studies, leaving the biological relevance of this degradation pathway uncertain in physiological contexts.

Bortezomib, a proteasome inhibitor, is a drug that reversibly inhibits the chymotrypsin-like activity of the β5 subunit, which constitutes the catalytic component of the proteasome. It has been clinically applied in the treatment of multiple myeloma and mantle cell lymphoma [16], and its efficacy is also being investigated in solid tumors such as breast and lung cancers [17]. However, the presence of bortezomib-refractory cases and the acquisition of resistance in tumor cells upon prolonged high-dose exposure remain major challenges [18]. Given the oncogenic role of PPM1D, PPM1D inhibitors have gained attention as potential therapeutic agents for malignant tumors. Several inhibitors, including GSK2830371 [19], SPI-001 [20], and CCT007093 [21], have been developed. Current efforts are focused on improving their bioavailability, stability, and selectivity, which may enhance their therapeutic efficacy in cancer treatment and support their progression to clinical trials.

In this study, we investigated the degradation pathways of PPM1D and found that PPM1D is directly degraded by the proteasome in a ubiquitin-independent manner. We also showed that PSMD14 and PSME3 act as activators in this process. Furthermore, the combined use of proteasome inhibitor and PPM1D inhibitor significantly enhanced the antitumor effect, potentially overcoming the reduced efficacy of proteasome inhibitor by accumulation of PPM1D. Our study introduces a novel, biologically relevant model substrate for future UbInPD studies. Additionally, this research identifies a potential new therapeutic target for cancer treatments based on proteasome inhibition.

## Methods

### Cell culture and reagents

All cells were maintained at 37 °C in a humidified atmosphere containing 5% CO_2_. HEK293T (RRID:CVCL_0063), HeLa (ATCC Cat# CCL-2, RRID:CVCL_0030), A549 (RRID:CVCL_0023), and HEK293 (RRID:CVCL_0045) cells were cultured in Dulbecco’s modified Eagle’s medium (Sigma-Aldrich, St. Louis, MO, USA). HCT116 (RRID:CVCL_0291) and U2OS (RRID:CVCL_0042) cells were grown in McCoy's 5A medium (Thermo Fisher Scientific, Waltham, MA, USA). U87MG (RRID:CVCL_0022) cells were maintained in Eagle’s minimum essential medium (Nacalai Tesque, Kyoto, Japan). hTERT-RPE1 (RRID:CVCL_4388) cells were maintained in Dulbecco’s modified Eagle medium/nutrient mixture F-12 (Thermo Fisher Scientific, Waltham, MA, USA). All media were supplemented with 10% fetal bovine serum (Thermo Fisher Scientific) and 1% penicillin/streptomycin (Nacalai Tesque).

The reagents used in this study included MG132 (474790, Merck, Darmstadt, Germany), carfilzomib (17554, Cayman Chemical, Ann Arbor, MI, USA), bafilomycin A1 (11038, Cayman Chemical), chloroquine (C6628, Sigma-Aldrich), bortezomib (SIH-328, StressMarq Biosciences, British Columbia, Canada), and GSK2830371 (S7573, Selleck Biotechnology, Kanagawa, Japan).

### Cloning of cDNAs and plasmid construction

Complementary DNAs encoding the human PPM1D gene were cloned into pGEX-6P-1 (GE Healthcare, Uppsala, Sweden). Wild-type PPM1D, PPM1D-N, PPM1D-C, PPM1D (450–605), PPM1D (502–605), PPM1D (396–570), and PPM1D (396–530), each with an N-terminal FLAG tag, were subcloned into the pQCXIP vector (Takara, Shiga, Japan). Wild-type NQO1 was subcloned into a modified pQCXIP vector (Takara) in which the puromycin resistance gene was replaced with a blasticidin resistance gene.

### Retrovirus expression system

Retroviral vectors were transfected into HEK293T cells together with the pCL10A1 vector (Novus Biologicals, Littleton, CO, USA) to generate recombinant retroviruses. HEK293T cells were infected with the recombinant retroviruses in the presence of polybrene (8 μg/ml, Sigma-Aldrich) and selected in a medium containing puromycin (5 μg/ml, Thermo Fisher Scientific) or blasticidin S (10 μg/ml, Fujifilm Wako, Osaka, Japan).

### Transfection and immunoblot analysis

Immunoblot analysis was performed with primary antibodies, horseradish peroxidase-conjugated secondary antibodies to anti-Mouse IgG antibody (Promega Cat# W4021, RRID:AB_430834, Madison, WI, USA, 1:15,000 dilution) or anti-Rabbit IgG antibody (Promega Cat# W4011, RRID:AB_430833, 1:15,000 dilution), and an enhanced chemiluminescence reagents (Thermo Fisher Scientific). The following primary antibodies were used: anti-FLAG M2 antibody (Sigma-Aldrich Cat# A8592, RRID:AB_439702, 1:2000 dilution), anti-PPM1D (Cell Signaling Technology, Danvers, MA, USA, 1:2000 dilution), anti-PSMD14 (Proteintech Cat# 12059-1-AP, RRID:AB_2170454, Rosemont, IL, USA, 1:2000 dilution), anti-PSME3 (Proteintech Cat# 14907-1-AP, RRID:AB_2171098, 1:2000 dilution), anti-c-Myc (Cell Signaling Technology Cat# 5605, RRID:AB_1903938, 1:2000 dilution), anti-NCOA4 (Bethyl Cat# A302-272A, RRID:AB_1850160, Montgomery, TX, USA, 1:2000 dilution), anti-vinculin (Sigma-Aldrich Cat# V9131, RRID:AB_477629, 1:50,000 dilution), anti-GAPDH (Thermo Fisher Scientific Cat# AM4300, RRID:AB_2536381, 1:100,000 dilution), anti-NCOA3/SRC3 (Cell Signaling Technology Cat# 2126, RRID:AB_823642, 1:2000 dilution), anti-CDKN1A/p21 (Cell Signaling Technology Cat# 2947, RRID:AB_823586, 1:2000 dilution), anti-TP53 (Cell Signaling Technology Cat# 2524, RRID:AB_331743, 1:2000 dilution), and anti-NQO1 (Proteintech Cat# 11451-1-AP, RRID:AB_2298729, 1:2000 dilution).

### Cycloheximide chase assay

Cycloheximide (50 μg/ml, Sigma-Aldrich) was added to the culture medium to inhibit translation and cell lysates were prepared at the indicated time points. The cell lysates were examined using immunoblot analysis. Densitometric analysis was carried out using NIH-ImageJ software.

### Sample preparation for mass spectrometry analysis

HEK293T cells (4.0 × 10^8^) stably expressing FLAG-tagged PPM1D-C were lysed in a solution containing 50 mM Tris–HCl (pH 7.6), 300 mM NaCl, 10% glycerol, 0.2% NP-40, 10 mM iodoacetamide (Sigma-Aldrich), 10 mM N-ethylmaleimide (Sigma-Aldrich), 0.5 mM 4-(2-aminoethyl)-benzenesulfonyl fluoride hydrochloride (AEBSF, Roche, Branchburg, NJ, USA), and 10 μM MG132 (Merck, Darmstadt, Germany). The cell lysates were sonicated and centrifuged at 20,000 × g for 10 min at 4 °C, and the resulting supernatant was incubated with anti-FLAG M2 agarose (Sigma-Aldrich) for 2 h at 4 °C. The resin was separated by centrifugation, washed five times with ice-cold lysis buffer and eluted twice with 50 μl of lysis buffer containing 0.25 mg/ml FLAG peptide (Sigma-Aldrich). Eluted peptides were dried by vacuum centrifugation, reduced with 55 mM dithiothreitol at 95 °C for 5 min (Thermo Fisher Scientific), alkylated with 10 mM iodoacetamide for 20 min at room temperature in the dark (Thermo Fisher Scientific), and digested overnight at 37 °C with 50 ng/μl trypsin (Promega, Madison, WI, USA) in the presence of 0.01% RapiGest SF (Waters, Milford, MA, USA). After tryptic digestion, the samples were acidified with TFA and purified by solid-phase extraction using GL-Tip GC and GL-Tip SDB (GL Sciences, Tokyo, Japan).

### Mass spectrometry analysis

Desalted tryptic digests were analyzed by nano-flow ultra-high-performance liquid chromatography (EASY-nLC 1000; Thermo Fisher Scientific) coupled online to an Orbitrap Elite instrument (Thermo Fisher Scientific). The mobile phases were 0.1% formic acid in water (solvent A) and 0.1% formic acid in 100% acetonitrile (solvent B). Peptides were directly loaded onto a Reprosil-Pur C18 analytical column (3 μm in particle size, 75 μm in inner diameter, and 12 cm in length; Nikkyo Technos, Tokyo, Japan) and separated using a 150-min two-step gradient (0–35% for 130 min, 35–100% for 5 min, and 100% for 15 min of solvent B) at a constant flow rate of 300 nl/min. For ionization, a liquid junction voltage of 1.6 kV and a capillary temperature of 200 °C were used. The Orbitrap Elite instrument was operated in the data-dependent MS/MS mode using Xcalibur software (RRID:SCR_014593, Thermo Fisher Scientific) with survey scans acquired at a resolution of 120,000 at m/z 400. The top 10 most abundant isotope patterns with a charge ranging from 2 to 4 were selected from the survey scans with an isolation window of 2.0 m/z and fragmented by collision-induced dissociation with normalized collision energies of 35. The maximum ion injection times for the survey and MS/MS scans were 60 ms, and the ion target values were set to 1 × 10⁶ for the survey and MS/MS scans.

### Protein identification from MS data

Proteome Discoverer software (version 2.4; RRID:SCR_014477; Thermo Fisher Scientific) was used to generate peak lists. The MS/MS spectra were searched against the UniProt Knowledgebase (version 2017_10; RRID:SCR_002380) using the SequestHT search engine. The precursor and fragment mass tolerances were set to 10 ppm and 0.6 Da, respectively. Methionine oxidation, protein amino-terminal acetylation, Asn/Gln deamidation, Ser/Thr/Tyr phosphorylation, diglycine modification of Lys side chains, and Cys carbamidomethyl modification were set as variable modifications for database searching. Peptide identification was filtered at a 1% false discovery rate. Label-free quantification was calculated using the intensities of precursor ions in the precursor ions quantifier node. Normalization was performed such that the sum of the abundance values for all peptides in each sample was the same. The log2 value of the abundance ratio (PPM1D-C versus Mock) was calculated for proteins related to proteasome subunit.

### RNA interference

Silencer Select siRNA for human PSMD14 (s19921 and s19919), human PSME3 (s19871 and s19872), and negative control (4390843) were purchased from Thermo Fisher Scientific. HEK293T cells were transfected with the siRNA using Lipofectamine RNAiMAX Transfection Reagent (Thermo Fisher Scientific).

### Recombinant protein purification

Recombinant human PPM1D protein was expressed in Escherichia coli strain DH5α harboring pGEX-6P-1-PPM1D for 16 h with 0.2 mM isopropyl-β-d-thiogalactopyranoside (Sigma-Aldrich). One liter of bacterial culture medium was centrifuged, and the pellet was resuspended in 40 ml of PBS (Thermo Fisher Scientific) supplemented with AEBSF (Roche, Branchburg, NJ) and disrupted with a French pressure cell (Aminco, Thermo Fisher Scientific). The lysates were clarified by ultracentrifugation, and the supernatants were incubated on ice for 2 h after the addition of 1 ml of glutathione sepharose 4B beads (GE Healthcare, Uppsala, Sweden). The beads were washed 5 times in PBS and the bound proteins were treated with PreScission protease (Cytiva, Marlborough, MA, USA) at 4 °C overnight. The excised GST and PreScission protease were removed from PPM1D by repurification on glutathione-Sepharose 4B beads.

### Degradation of PPM1D protein by 20S proteasomes

Human 20S proteasome protein was purchased from Boston Biochem (Cambridge, MA, USA). The 20S proteasome (0.25 μg) was incubated with 2 ng PPM1D protein for 1 h at 30 °C in a buffer containing 50 mM Tris–HCl, pH 7.5, 5 mM MgCl_2_, 1 mM DTT, and 0.2 mg/ml BSA. Products were analyzed by SDS-PAGE and immunoblotting.

### Cell viability assay

Cell viability was evaluated using the Cell Counting Kit-8 (Dojindo Molecular Technologies, Inc., Kumamoto, Japan). 1 × 10^4^ cells in 100 µl of medium were seeded into each well of a 96-well plate and incubated at 37 °C for 48 h with the indicated concentrations of bortezomib, GSK2830371, or both. After incubation, 10 µl of Cell Counting Kit-8 reagent was added to each well, and the cells were incubated for an additional 1 h at 37 °C. The absorbance was then measured at 450 nm using a SpectraMax Paradigm (Molecular Devices, San Jose, CA, USA). The cell viability rate was calculated as a percentage of the control based on samples without drug exposure.

### Evaluation of drug combination effects

To evaluate the combination effects of bortezomib and GSK2830371, cell viability data were analyzed using CompuSyn software (ComboSyn Inc., Paramus, NJ, USA), based on the Chou–Talalay method [22]. Drug concentrations were applied at a fixed molar ratio derived from the IC_50_ values of each single agent. The combination index (CI) was calculated at different effect levels (fraction affected, Fa), where CI < 1 indicates synergism, CI = 1 indicates an additive effect, and CI > 1 indicates antagonism. Median-effect plots CI–Fa plots, and isobolograms were generated automatically by the software.

### Establishment of bortezomib-resistant cells

To establish bortezomib-resistant cell Lines, parental cells were continuously cultured for 30 days in medium containing bortezomib at concentrations ranging from 10 to 100 nM, increasing in 5 nM increments. Cells were passaged every 2 to 3 days to maintain sub-confluent growth. After 30 days, cells that survived in the highest bortezomib concentration were collected as the resistant population. Bortezomib resistance was evaluated using a cell viability assay, in the same manner as for the parental cells, by determining the IC_50_ values after 48 h of bortezomib treatment. Resistance was confirmed by comparing the IC_50_ values between the resistant and parental cell lines.

### Statistical analysis

All experiments were performed at least three independent times or at least twice in triplicate. Data are presented as mean ± standard deviation (SD). The unpaired Student's t-test was used to compare differences between experimental and control groups. Differences with P < 0.05 were considered statistically significant. All analyzes were performed using Microsoft Excel (RRID:SCR_016137).

## Results

### PPM1D is a short-lived protein

**Fig. 1:**
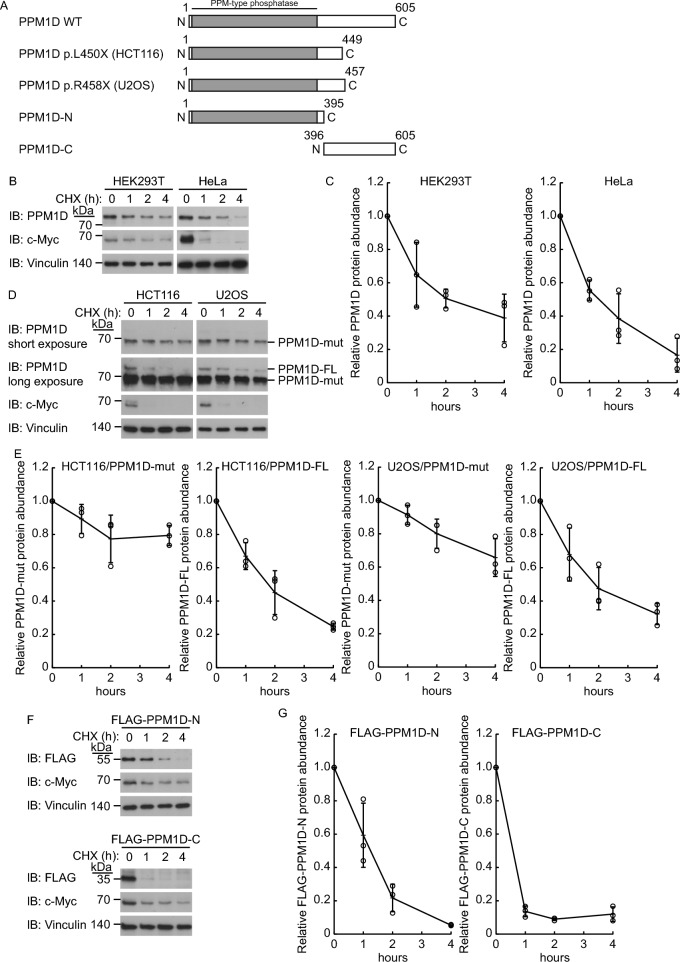
PPM1D is a short-lived protein, and its C-terminal region influences its stability. **A** Schematic representation of wild-type and mutant PPM1D constructs used in this study. **B**, **C** Cycloheximide-chase assays of HEK293T cells and HeLa cells (n = 3). Representative immunoblots (**B**) and quantification (**C**) of PPM1D protein levels are shown. Cells were cultured in the presence of cycloheximide (50 μg/ml) for the indicated times. Protein levels were normalized to vinculin. **D**, **E** Cycloheximide-chase assays of HCT116 cells and U2OS cells (n = 3). Representative immunoblots (**D**) and quantification (**E**) of PPM1D mutant (PPM1D-mut) or PPM1D full-length (PPM1D-FL) protein levels are shown. Cells were cultured in the presence of cycloheximide (50 μg/ml) for the indicated times. Protein levels were normalized to vinculin. **F**, **G** Cycloheximide-chase assays of HEK293T cells with stable expression of FLAG-PPM1D-N or FLAG-PPM1D-C (n = 3). Representative immunoblots (**F**) and quantification (**G**) of PPM1D mutant protein levels are shown. Cells were cultured in the presence of cycloheximide (50 μg/ml) for the indicated times. Protein levels were normalized to vinculin. Data represent the mean ± SD

To investigate the intracellular stability of PPM1D, we performed a cycloheximide chase assay using HEK293T and HeLa cells, which express wild-type PPM1D (Fig. 1B, C). The results showed that wild-type PPM1D was rapidly degraded in cells. HCT116 and U2OS cells harbor heterozygous mutations in the PPM1D gene, resulting in the expression of both wild-type PPM1D and a C-terminal truncation mutant of PPM1D. Previous studies have reported that the C-terminal truncation mutant of PPM1D exhibits enhanced stability compared to its wild-type counterpart [4]. We reevaluated and confirmed that the C-terminal truncation mutant of PPM1D exhibits increased stability compared to wild-type PPM1D in cells (Fig. 1A, D, E). Furthermore, to verify the contribution of specific PPM1D regions to protein stability, we established HEK293T cell lines exogenously expressing either the N-terminal region (FLAG-PPM1D-N) or the C-terminal region (FLAG-PPM1D-C) of PPM1D. Cycloheximide chase assays revealed that FLAG-PPM1D-C was degraded more rapidly than wild-type PPM1D (Fig. 1F, G).

### PPM1D undergoes proteasome-dependent degradation

**Fig. 2:**
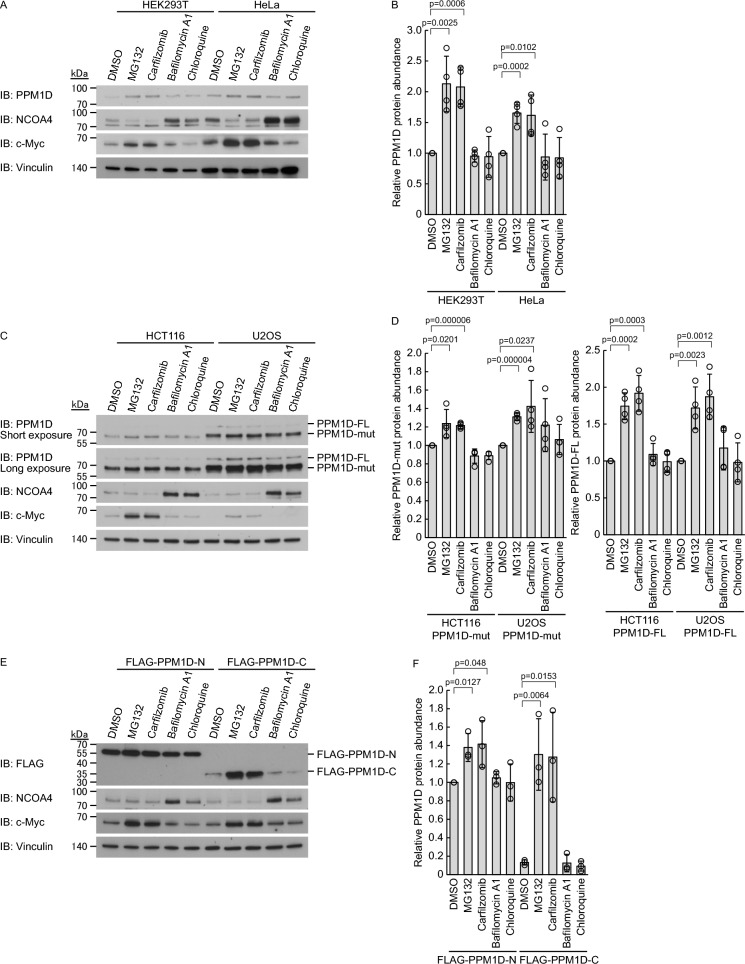
PPM1D undergoes proteasome-dependent degradation. **A**, **B** HEK293T cells and HeLa cells were treated for 3 h with inhibitors: MG132 (10 μM), Carfilzomib (10 μM), Bafilomycin A1 (100 nM), and Chloroquine (50 μM) using DMSO as vehicle control (n = 3). Representative immunoblots (**A**) and quantification (**B**) of PPM1D protein levels are shown. Protein levels were normalized to vinculin. **C**, **D** HCT116 cells and U2OS cells were treated for 3 h with inhibitors: MG132 (10 μM), carfilzomib (10 μM), bafilomycin A1 (100 nM), and chloroquine (50 μM) using DMSO as vehicle control. Representative immunoblots (**C**) and quantification (**D**) of PPM1D mutant (PPM1D-mut) or PPM1D full-length (PPM1D-FL) protein levels are shown. Protein levels were normalized to vinculin. **E, F** HEK293T cells with stable expression of FLAG-PPM1D-N or FLAG-PPM1D-C (n = 3) were treated for 3 h with inhibitors: MG132 (10 μM), carfilzomib (10 μM), bafilomycin A1 (100 nM), and chloroquine (50 μM) using DMSO as vehicle control. Representative immunoblots (**E**) and quantification (**F**) of PPM1D mutant protein levels are shown. Protein levels were normalized to vinculin. Data represent the mean ± SD

To determine whether PPM1D undergoes degradation via the proteasome or lysosome, we treated various cell lines with proteasome inhibitors (MG132 and carfilzomib) and lysosome inhibitors (bafilomycin A1 and chloroquine) and measured changes in PPM1D expression levels. As controls, we confirmed that NCOA4, which is degraded through the lysosomal pathway, accumulated upon lysosome inhibition, whereas c-Myc, which undergoes proteasomal degradation via both ubiquitin-dependent and ubiquitin-independent mechanisms, accumulated in response to proteasome inhibition [8, 23, 24]. In HEK293T and HeLa cells expressing wild-type PPM1D, treatment with proteasome inhibitors led to the accumulation of wild-type PPM1D, whereas lysosome inhibitors had no effect (Fig. 2A, B). We further examined HCT116 and U2OS cells and found that proteasome inhibition similarly resulted in the accumulation of wild-type PPM1D, while the C-terminal truncation mutant showed a more modest accumulation. In contrast, lysosome inhibitors did not affect the levels of either protein (Fig. 2C, D). In HEK293T cells overexpressing either FLAG-PPM1D-N or FLAG-PPM1D-C, proteasome inhibition led to a moderate accumulation of FLAG-PPM1D-N and a substantial accumulation of FLAG-PPM1D-C, whereas lysosome inhibitors had no effect on either variant (Fig. 2E, F). These findings indicate that PPM1D degradation is regulated in a proteasome-dependent manner, with the C-terminal region playing a particularly important role in this process.

### PPM1D is degraded via distinct N- and C-terminal pathways, with C-terminal ubiquitination selectively inhibiting the C-terminal, ubiquitin-independent mechanism

**Fig. 3:**
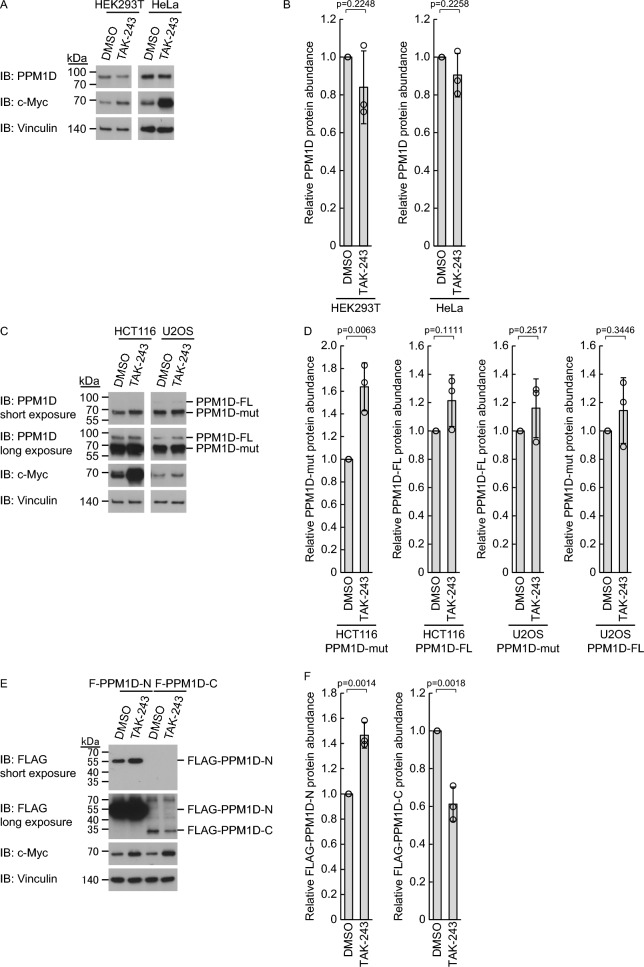
C-terminal region of PPM1D mediates ubiquitin-independent proteasomal degradation. **A**, **B** HEK293T cells and HeLa cells were treated for 3 h with TAK-243 (250 nM) using DMSO as vehicle control (n = 3). Representative immunoblots (**A**) and quantification (**B**) of PPM1D protein levels are shown. Protein levels were normalized to vinculin. **C**, **D** HCT116 cells and U2OS cells were treated for 3 h with TAK-243 (250 nM) using DMSO as vehicle control (n = 3). Representative immunoblots (**C**) and quantification (**D**) of PPM1D mutant (PPM1D-mut) or PPM1D full-length (PPM1D-FL) protein levels are shown. Protein levels were normalized to vinculin. **E**, **F** HEK293T cells with stable expression of FLAG-PPM1D-N or FLAG-PPM1D-C (n = 3) were treated for 3 h with TAK-243 (250 nM) using DMSO as vehicle control (n = 3). Representative immunoblots (**E**) and quantification (**F**) of PPM1D mutant protein levels are shown. Protein levels were normalized to vinculin. Data represent the mean ± SD

**Fig. 4:**
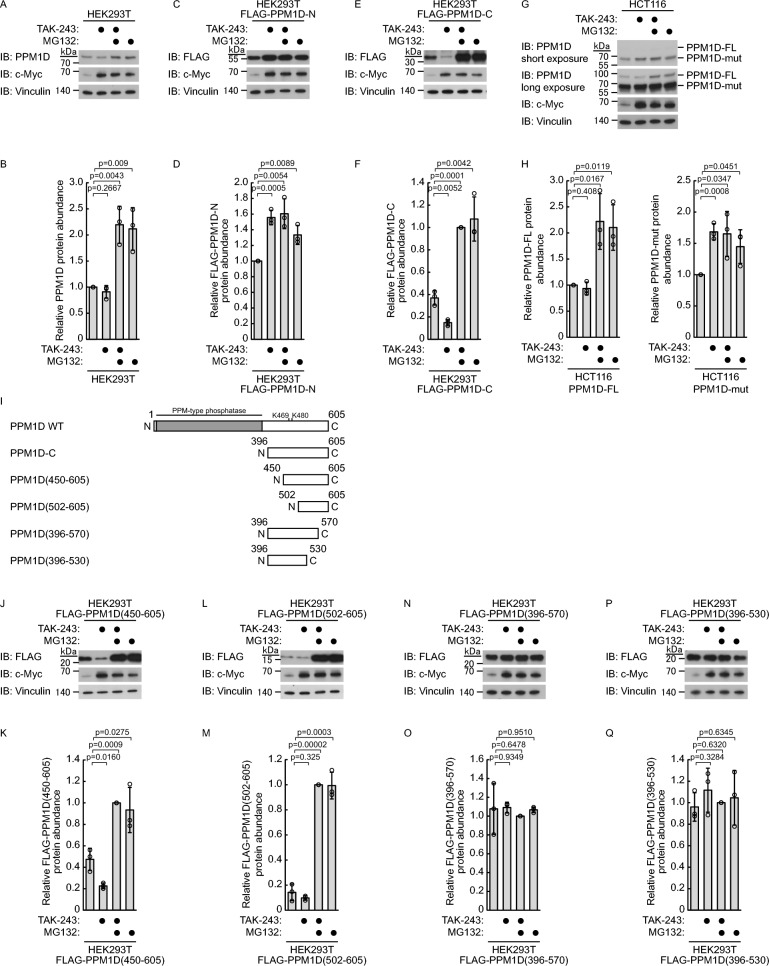
Co-treatment with TAK-243 and MG132 identified critical regions of PPM1D involved in the regulation of both ubiquitin-dependent and ubiquitin-independent protein degradation. HEK293T or HCT116 cells were treated with TAK-243 (250 nM) and/or MG132 (10 μM) for 3 h, with DMSO as vehicle control (n = 3). Representative immunoblots (**A**, **C**, **E**, **G**, **J**, **L**, **N**, **P**) and quantification (**B**, **D**, **F**, **H**, **K**, **M**, **O**, **Q**) of PPM1D protein levels are shown. **A**–**B** Endogenous PPM1D in HEK293T cells. **C**–**F** HEK293T cells stably expressing FLAG-tagged PPM1D-N or PPM1D-C mutants. **G**–**H** HCT116 cells expressing PPM1D full-length (PPM1D-FL) or mutant (PPM1D-mut). **I** Schematic representation of wild-type and mutant PPM1D constructs used in this study. **J**–**Q** HEK293T cells stably expressing various FLAG-tagged PPM1D truncation mutants (450–605, 502–605, 396–570, 396–530). Protein levels were normalized to vinculin. Data represent the mean ± SD

We next investigated whether the proteasome-mediated degradation of PPM1D is dependent on ubiquitination. We treated each cell line with the E1 ubiquitin-activating enzyme inhibitor TAK-243 and measured changes in PPM1D levels. In HEK293T and HeLa cells, wild-type PPM1D levels remained unchanged upon E1 inhibition (Fig. 3A, B). In HCT116 and U2OS cells, wild-type PPM1D was also unaffected, whereas the C-terminal truncation mutant exhibited an increase only in HCT116 cells, suggesting the stability of the C-terminal truncation mutant of PPM1D in HCT116 is also partially affected by ubiquitin-dependent degradation (Fig. 3C, D). In HEK293T cells overexpressing FLAG-PPM1D-N or FLAG-PPM1D-C, E1 inhibition resulted in a gradual accumulation of FLAG-PPM1D-N, whereas FLAG-PPM1D-C decreased rather than accumulated (Fig. 3E, F). We further measured the extent of proteasome-mediated degradation under conditions where ubiquitination was inhibited by simultaneous treatment with TAK-243 and MG132. In HEK293T cells, the level of wild-type PPM1D, which did not change upon E1 inhibition alone, accumulated to a similar extent as with MG132 single treatment upon combined inhibition with TAK-243 and MG132 (Fig. 4A, B). This suggests that the degradation of wild-type PPM1D primarily depends on ubiquitin-independent proteasomal degradation, or that the amount of ubiquitin-dependent degradation is balanced by a compensatory increase in ubiquitin-independent degradation upon ubiquitination inhibition. In HEK293T cells overexpressing FLAG-PPM1D-N, the accumulation of FLAG-PPM1D-N induced by E1 inhibition alone was not further increased by the combined inhibition (Fig. 4C, D), indicating that the degradation of FLAG-PPM1D-N mainly occurs in a ubiquitin-dependent manner. In HEK293T cells overexpressing FLAG-PPM1D-C, the decrease in FLAG-PPM1D-C caused by E1 inhibition alone was restored to levels similar to MG132 single treatment by the combined inhibition (Fig. 4E, F). This indicates that ubiquitination inhibition leads to a compensatory enhancement of ubiquitin-independent proteasomal degradation. In HCT116 cells, wild-type PPM1D showed changes similar to those in HEK293T cells, and the C-terminal deletion mutant exhibited changes similar to FLAG-PPM1D-N (Fig. 4G, H), indicating that the degradation of the C-terminal deletion mutant mainly occurs in a ubiquitin-dependent manner. We further introduced a series of truncated constructs derived from PPM1D-C into HEK293T cells and performed the same experimental analyses (Fig. 4I). PPM1D (450–605) exhibited a reduction in protein levels upon E1 inhibition, similar to PPM1D-C, and this reduction was rescued to a comparable extent by co-treatment with MG132 (Fig. 4J, K). In contrast, PPM1D (502–605) did not show any decrease upon E1 inhibition, suggesting that the region encompassing residues 450–501 is required for ubiquitination-dependent suppression of ubiquitin-independent proteasomal degradation (Fig. 4L, M). Moreover, PPM1D (396–570) and PPM1D (396–530) did not respond to either E1 or proteasome inhibition, indicating that the C-terminal 35 amino acids of PPM1D are essential for ubiquitin-independent proteasomal degradation (Fig. 4N–Q). In summary, wild-type PPM1D undergoes both ubiquitin-dependent degradation mediated by its N-terminal region and ubiquitin-independent proteasomal degradation mediated by its C-terminal 35 amino acids. Additionally, the region spanning residues 450–501 is necessary for ubiquitination-mediated suppression of the ubiquitin-independent proteasomal degradation pathway.

### PPM1D is directly degraded by the 20S proteasome

**Fig. 5:**
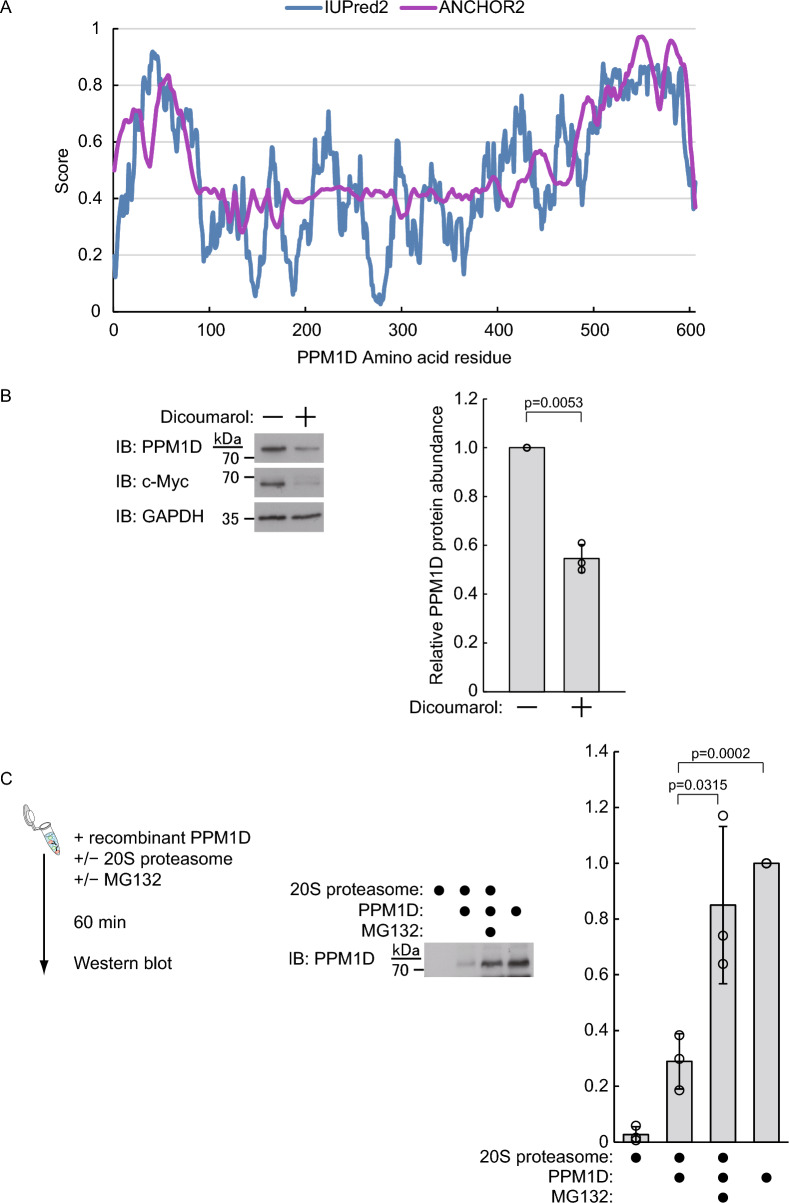
The 20S proteasome directly recognizes and degrades PPM1D in a ubiquitin-independent manner. **A** Two methods were performed to assess the IDR regions of PPM1D: the blue line indicates IUPred2 and the purple line indicates ANCHOR2. Regions above 0.5 represent IDR regions. **B** HEK293T cells were treated for 3 h with dicoumarol (300 μM) (n = 3). Representative immunoblots and quantification of PPM1D protein levels are shown. Protein levels were normalized to GAPDH. **C** Purified PPM1D and 20S proteasome were incubated in the presence or absence of MG132 (10 μM) for 60 min (n = 3). An aliquot of the reaction mixture was subsequently analyzed by immunoblotting using an anti-PPM1D antibody. Protein levels were normalized to control. Data represent the mean ± SD

Since the degradation of PPM1D via its C-terminus was found to be ubiquitin-independent, we first hypothesized that PPM1D may be directly degraded by the 20S proteasome. The 20S proteasome is known to recognize and degrade proteins independently of ubiquitination by targeting intrinsically disordered regions (IDRs) [25]. Using IUPred2A [26], we predicted IDRs within the PPM1D amino acid sequence and found that the C-terminal region beyond approximately residue 500 is likely to be intrinsically disordered and possibly functions as a degron for the stability of PPM1D (Fig. 5A). It has been reported that ubiquitin-independent degradation by the 20S proteasome is partially inhibited by NAD(P)H:quinone oxidoreductase-1 (NQO1) [27]. To verify the role of the 20S proteasome in PPM1D degradation, we treated cells with the NQO1 inhibitor dicoumarol. As a result, wild-type PPM1D levels decreased upon dicoumarol treatment (Fig. 5B), suggesting that NQO1 inhibition enhances 20S proteasome activity, and leading to increased PPM1D degradation. We also treated HEK293T cells overexpressing FLAG-PPM1D-N with dicoumarol and observed a decrease in FLAG-PPM1D-N levels (Supplementary Fig. S1A, B), suggesting that enhanced 20S proteasome activity can promote degradation of PPM1D lacking the C-terminus; however, as shown in Fig. 4C and D, it should be noted that under normal conditions, FLAG-PPM1D-N is primarily degraded in a ubiquitin-dependent manner. We also examined whether NQO1 overexpression affects the stability of PPM1D and FLAG-PPM1D-C. In HEK293T cells overexpressing NQO1, no changes were observed in the levels of wild-type PPM1D, c-Myc, or p53—other known 20S proteasome substrates—indicating that endogenous NQO1 is present at sufficient levels to suppress 20S proteasome activity in these cells (Supplementary Fig. S1C, D). Similar results were obtained in HEK293T cells stably expressing FLAG-PPM1D-C (Supplementary Fig. S1E, F). We next performed an in vitro degradation assay using recombinant PPM1D and purified 20S proteasome and found that PPM1D levels decreased upon incubation with the 20S proteasome (Fig. 5C). Taken together, these results demonstrate that PPM1D is directly recognized and degraded by the 20S proteasome in a ubiquitin-independent manner.

### Proteasome activator PSMD14 and PSME3 promote proteasomal degradation of PPM1D

**Fig. 6:**
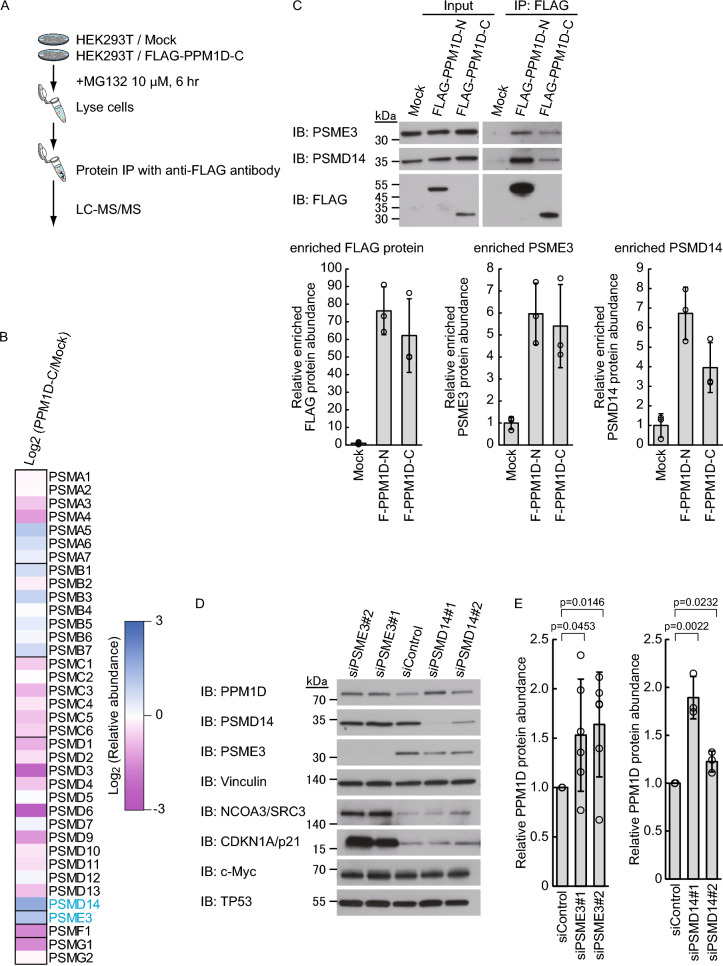
Proteasome activators PSMD14 and PSME3 promote proteasomal degradation of PPM1D. **A** Schematic representation of the binding protein identification workflow. FLAG-PPM1D-C-interacting proteins were immunoprecipitated from HEK293T cells treated with 10 μM MG132 for 6 h. The immunoprecipitated proteins were subsequently identified using LC–MS/MS analysis, followed by label-free quantification. **B** The 19S proteasome regulatory subunit PSMD14 and the proteasome activator PSME3 were identified as putative interaction partners of FLAG-PPM1D-C. PPM1D-C-interacting proteins associated with proteasome subunits are presented. The heatmap depicts the relative abundance of each protein compared to control cells. **C** In vivo interaction of PSMD14 and PSME3 with FLAG-PPM1D-C was assessed by co-immunoprecipitation (n = 3). Whole cell lysates from HEK293T cells stably expressing FLAG-PPM1D-C were subjected to immunoprecipitation using an anti-FLAG antibody. The immunoprecipitates were then analyzed by immunoblotting with antibodies against FLAG, PSMD14, and PSME3. **D**, **E** HEK293T cells were transfected with siRNAs targeting PSME3 (n = 6) and PSMD14 (n = 3). After 48 h, cells were harvested and subjected to immunoblot analysis using antibodies against PPM1D, PSME3, PSMD14, NCOA3/SRC3, CDKN1A/p21, c-Myc, and TP53. Representative immunoblots (**D**) and quantification (**E**) of PPM1D protein levels are shown. Protein levels were normalized to vinculin. Data represent the mean ± SD

We identified that PPM1D is directly targeted for degradation by the 20S proteasome. However, the reduction in wild-type PPM1D levels upon dicoumarol treatment was limited to approximately 50%, which was more gradual compared to that of c-Myc (Fig. 5B). Given that NQO1 has been reported to physically associate with the 20S proteasome but not with the 26S proteasome, it is plausible that dicoumarol specifically induces the degradation of UbInPD substrates via the 20S proteasome [10]. Considering the possibility that other proteasome-associated regulatory factors might be involved, we attempted to identify proteins that bind to the C-terminus of PPM1D. To achieve this, HEK293T cells overexpressing FLAG-PPM1D-C and corresponding control cells were treated with MG132, followed by immunoprecipitation using an anti-FLAG antibody and liquid chromatography-tandem mass spectrometry analysis (Fig. 6A). As a result, PSMD14, a subunit of the 19S regulatory particle, and PSME3, a proteasome activator, were identified as candidate molecules interacting with FLAG-PPM1D-C (Fig. 6B). Consistent with the mass spectrometry results, immunoblotting also confirmed that PSMD14 and PSME3 co-precipitated with FLAG-PPM1D-C (Fig. 6C). To investigate the effects of PSMD14 and PSME3 on PPM1D degradation, we performed knockdown experiments in HEK293T cells. Knockdown of either PSMD14 or PSME3 resulted in the stabilization of wild-type PPM1D (Fig. 6D, E). Consistent with previous reports, accumulation of SRC-3 [28] and p21/CDKN1A [29] was observed upon PSME3 knockdown, whereas c-Myc [8] and p53 [10], which undergo ubiquitin-independent proteasomal degradation regardless of PSME3 or PSMD14, were unaffected by the knockdown. We also examined the effect of PSME3 and PSMD14 knockdown on FLAG-PPM1D-C in HEK293T cells. Knockdown of PSMD14 resulted in a clear accumulation of FLAG-PPM1D-C, whereas knockdown of PSME3 had no apparent effect (Supplementary Fig. S2A, B). Furthermore, when we performed the same experiment using cells expressing FLAG-PPM1D-N, knockdown of neither PSMD14 nor PSME3 affected its protein level (Supplementary Fig. S2C, D). Taken together, these results suggest that degradation of the C-terminal region of PPM1D (PPM1D-C) is promoted via a PSMD14-dependent pathway, whereas PSME3-dependent degradation may require the presence of full-length PPM1D.

### Simultaneous inhibition of proteasome and PPM1D can effectively inhibit malignant tumor progression

**Fig. 7:**
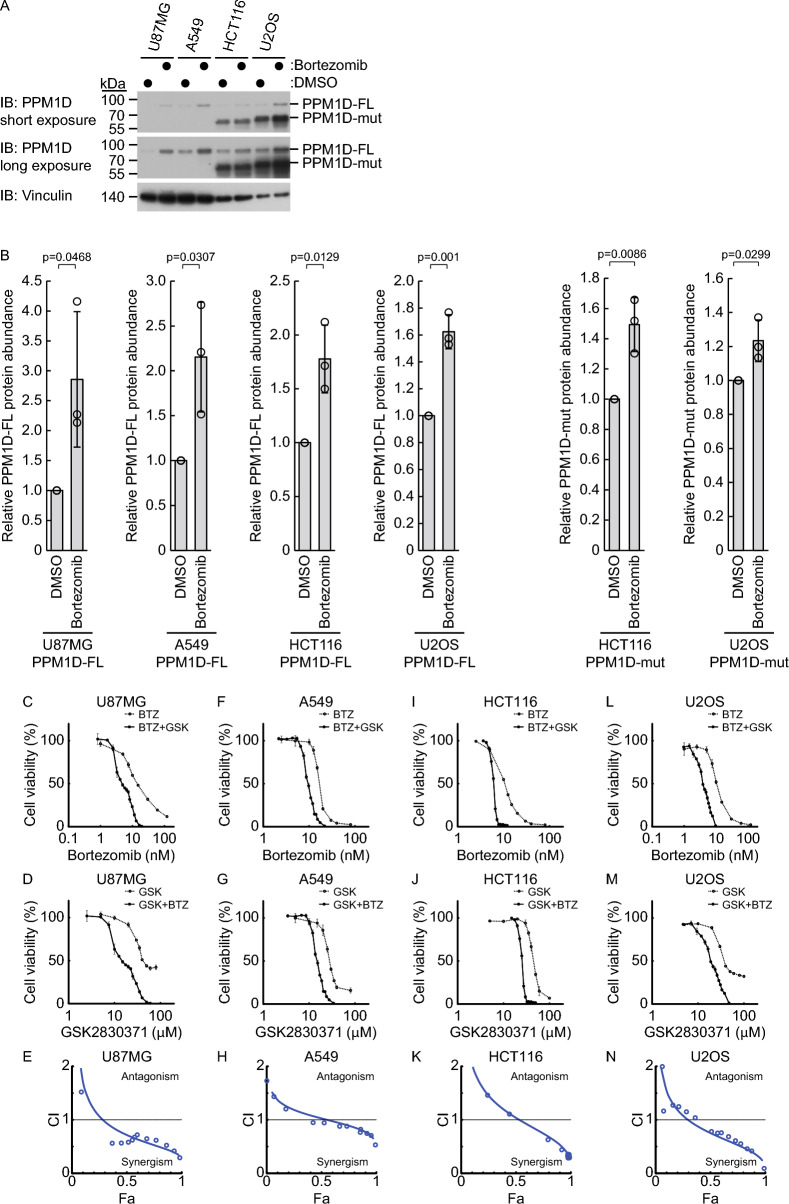
The tumor-suppressing potency of proteasome inhibitor is enhanced through combination with PPM1D inhibitor. **A**, **B** Cells were treated for 8 h with bortezomib: U87MG (64 nM), A549 (32 nM), HCT116 (16 nM), and U2OS (32 nM) using DMSO as vehicle control (n = 3). Representative immunoblots (**A**) and quantification (**B**) of PPM1D mutant (PPM1D-mut) or PPM1D full-length (PPM1D-FL) protein levels are shown. Protein levels were normalized to vinculin. **C**–**N** Cells were treated for 48 h with various concentrations of bortezomib, GSK2830371, or their combination at a fixed molar ratio based on the IC_50_ values of each drug. Cell viability was assessed using the Cell Counting Kit-8 assay (n = 3). Dose–response curves for bortezomib alone and in combination with GSK2830371 are shown in panels **C**, **F**, **I**, and **L**, while those for GSK2830371 alone and in combination with bortezomib are shown in panels **D**, **G**, **J**, and **M**. Data represent mean ± SD. Combination Index (CI) plots (**E**, **H**, **K**, and **N**), extracted from CompuSyn reports, depict the relationship between the fraction affected (Fa, x-axis) and CI (y-axis). CI values < 1 indicate synergistic interaction between the two drugs

Proteasome inhibitors are used as therapeutic agents for malignant tumors. Since PPM1D functions as an oncogenic factor [2], its accumulation upon proteasome inhibition may attenuate the therapeutic efficacy of proteasome inhibitors. We hypothesized that the combination of the proteasome inhibitor bortezomib and the PPM1D inhibitor GSK2830371 could effectively suppress malignant tumor progression. To test this, we performed a cell viability assay using U87MG, A549, HCT116, and U2OS cells, all of which harbor wild-type p53. Consistent with our previous findings, bortezomib treatment led to PPM1D accumulation in these cells (Fig. 7A, B). Co-treatment with bortezomib and GSK2830371 resulted in greater suppression of cell proliferation compared with either agent alone in all tested p53 wild-type cell lines (Fig. 7C, D, F, G, I, J, L, and M). To quantitatively assess the interaction between the two drugs, we performed combination index analysis using the Chou–Talalay method. This analysis demonstrated a synergistic effect (CI < 1) in all cell lines at Fa levels of at least 0.5 (Fig. 7E, H, K, and N), indicating that the combination of proteasome and PPM1D inhibition enhances the anticancer efficacy relative to monotherapy. The corresponding median-effect plots and isobolograms for U87MG, A549, HCT116, and U2OS cells are presented in Supplementary Fig. S3A–H. To examine whether the synergistic effect of bortezomib and GSK2830371 is specific to cancer cells, we conducted similar combination experiments using immortalized non-tumorigenic cell lines, hTERT-RPE1 and HEK293. In hTERT-RPE1 cells, the combination showed a synergistic effect comparable to that observed in tumor cells (Supplementary Fig. S4A–E). In contrast, HEK293 cells did not exhibit synergy at Fa < 0.9 (Supplementary Fig. S4F–J), suggesting that the combination effect may be cell type-dependent.

### Proteasome and PPM1D inhibition exhibits synergistic effects even in bortezomib-resistant cells

**Fig. 8:**
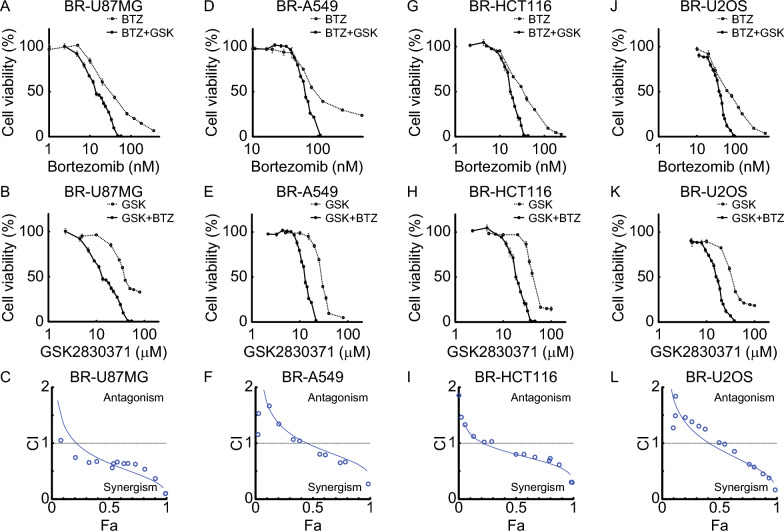
Bortezomib and GSK2830371 exhibit synergistic cytotoxicity in bortezomib-resistant cells. Bortezomib-resistant (BR) cells were treated for 48 h with various concentrations of bortezomib, GSK2830371, or their combination at a fixed molar ratio based on the IC_50_ values of each drug. Cell viability was assessed using the Cell Counting Kit-8 assay (n = 3). Dose–response curves are shown in panels **A**, **B**, **D**, **E**, **G**, **H**, **J**, and **K**, comparing single-drug treatments with the combination. Data represent mean ± SD. Combination index (CI) plots extracted from CompuSyn reports are shown in panels **C**, **F**, **I**, and **L**. The x-axis represents the fraction affected (Fa), and the y-axis indicates the CI value. CI < 1 denotes synergism between the two drugs

To assess whether the combination effect of bortezomib and GSK2830371 is retained in drug-resistant cells, we established bortezomib-resistant derivatives of U87MG, A549, HCT116, and U2OS cell lines by continuous exposure to bortezomib-containing medium for 30 days. The concentrations of bortezomib used during selection, along with the IC₅₀ values for bortezomib and GSK2830371 in both parental and resistant cells, are summarized in Table 1. Despite acquired resistance, all resistant cell lines exhibited a similar pattern to their parental counterparts: co-treatment with bortezomib and GSK2830371 resulted in greater inhibition of cell proliferation than either agent alone (Fig. 8A, B, D, E, G, H, J, and K). Furthermore, combination index analysis using the Chou–Talalay method demonstrated synergistic effects in all resistant Lines at Fa levels of at least 0.5 (Fig. 8C, F, I, and L). The corresponding median-effect plots and isobolograms for these resistant cell lines are shown in Supplementary Fig. S5A–H. These findings indicate that dual inhibition of the proteasome and PPM1D remains an effective strategy even in the setting of bortezomib resistance.

**Table 1:** Summary of IC_50_ values and resistance status in parental and bortezomib-resistant cell lines. Parental and bortezomib-resistant cell lines of U87MG, A549, HCT116, and U2OS were assessed for their sensitivity to bortezomib and GSK2830371 using cell viability assays. The resistant cell lines were generated by continuous culture in medium containing increasing concentrations of bortezomib, as shown

	U87MG	A549	HCT116	U2OS
Bortezomib IC_50_ (Parent, nM)	16.4	20.2	12.0	9.7
GSK2830371 IC_50_ (Parent, μM)	50.0	31.7	49.9	47.3
Bortezomib concentration for resistance induction (nM)	25.0	75.0	50.0	50.0
Bortezomib IC_50_ (Resistant, nM)	47.7	145.4	43.1	74.8
Bortezomib IC_50_ Fold (Resistant/Parent)	2.91	7.20	3.59	7.71
GSK2830371 IC_50_ (Resistant, μM)	45.2	29.0	45.9	31.5
GSK2830371 IC_50_ Fold (Resistant/Parent)	0.90	0.91	0.92	0.67

## Discussion

**Fig. 9:**
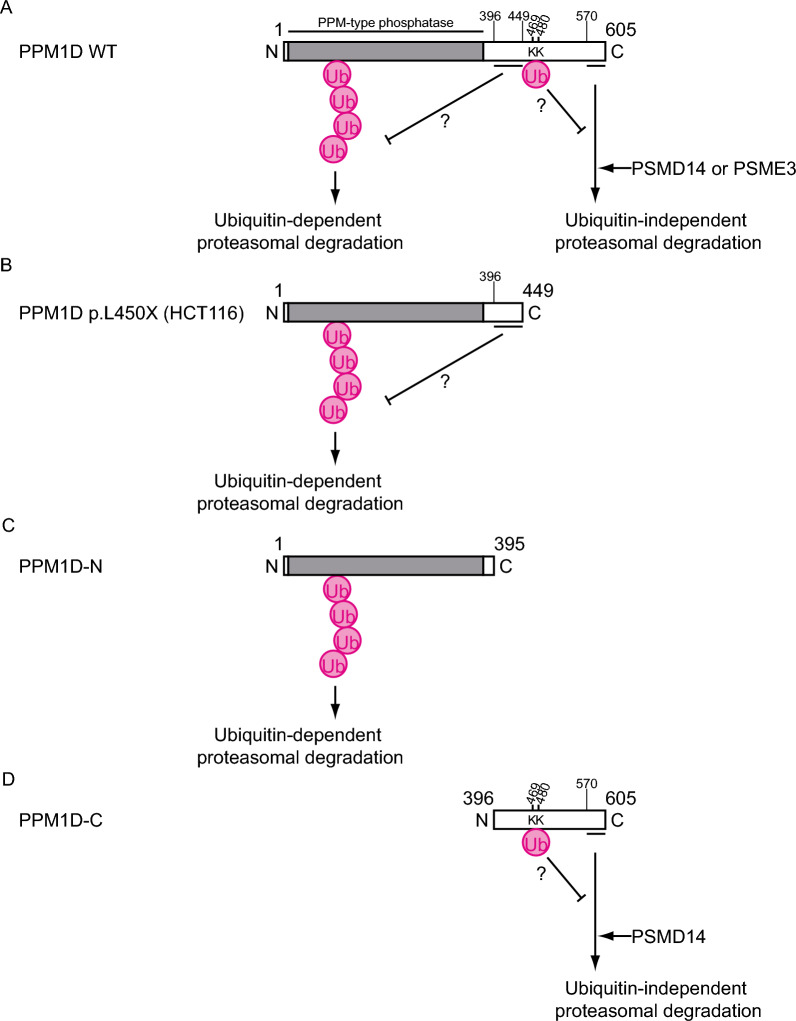
Schematic illustration of the mechanism regulating PPM1D protein stability. Schematic of wild-type PPM1D is shown in **A**; that of the HCT116 mutant (p.L450X) in **B**; PPM1D-N in **C**; and PPM1D-C in **D**

In this study, we demonstrated that the 20S proteasome directly targets and degrades PPM1D by recognizing its C-terminal IDR as a degron. Interestingly, the region spanning residues 450–501 of PPM1D was found to be essential for ubiquitination-mediated suppression of UbInPD. We also provide evidence suggesting that PSMD14 and PSME3 may play activating roles in this process (Fig. 9A). Moreover, we hypothesized that PPM1D accumulation upon proteasome inhibition contributes to chemoresistance in tumor cells and provided evidence that the combination of a proteasome inhibitor and a PPM1D inhibitor enhances antitumor efficacy.

HCT116 (p.L450X) and U2OS (p.R458X) cells heterozygously express both wild-type and C-terminally truncated mutant forms of PPM1D, with the mutant proteins exhibiting marked stabilization. This stabilization is likely due to their escape from UbInPD-mediated degradation, which normally targets the C-terminus of the protein. In contrast, the degradation rate of the engineered mutant FLAG-PPM1D (1–395) was higher than that of the endogenous mutants in HCT116 and U2OS cells (Fig. 1E, G). Given that both FLAG-PPM1D (1–395) and the HCT116 mutant (p.L450X) are primarily degraded via the ubiquitin–proteasome system (Fig. 4C, D, G, and H), it is possible that the region encompassing amino acid residues 396–449 may interfere with recognition by ubiquitin ligases (Fig. 9B, C).

Ubiquitination-mediated suppression of UbInPD may occur via two possible mechanisms. One possibility is an indirect mechanism, in which ubiquitinated non-PPM1D proteins interfere with PPM1D degradation by either competing for the degradation machinery or promoting the ubiquitin-dependent degradation of factors that facilitate PPM1D UbInPD. The other possibility is a direct mechanism, in which ubiquitination of the region spanning residues 450–501 of PPM1D suppresses its UbInPD. This region contains lysine residues at positions 469 and 480, and it is conceivable that ubiquitination at these sites stabilizes the C-terminal structure of PPM1D, thereby inhibiting its recognition or processing by the proteasome (Fig. 9D).

UbInPD has been reported to occur through several mechanisms. These include direct recognition of target proteins by 20S proteasome subunits, recruitment mediated by regulatory particles such as PSME3 (Proteasome Activator 28γ) and Proteasome Activator 200, and involvement of shuttle factors like Rad23, ubiquilin, and midnolin [12, 30–32]. The 20S proteasome is also known to recognize misfolded or aberrantly structured proteins and IDRs [25, 33]. In this study, we demonstrated that PPM1D undergoes direct degradation by the 20S proteasome through experiments using the NQO1 inhibitor and in vitro assays with recombinant proteins. A part of the PPM1D degradation mechanism appears to depend on the direct recognition of an IDR located in the C-terminal portion of PPM1D by the 20S proteasome. On the other hand, the reduction in wild-type PPM1D levels following dicoumarol treatment was more gradual compared to that of c-Myc (Fig. 5B). This observation led us to hypothesize the involvement of additional cofactors in the UbInPD process of PPM1D, prompting us to identify PSMD14 and PSME3 as PPM1D C-terminal binding proteins. Indeed, the knockdown of PSMD14 and PSME3 resulted in PPM1D stabilization, confirming their role in promoting its degradation. PSMD14 is a subunit of the 19S proteasome, while PSME3 is a proteasome activator. Notably, PSME3 is involved in promoting the degradation of proteins associated with cell proliferation and the cell cycle [34]. It has also been reported to recruit and facilitate the ubiquitin-independent degradation of the transcriptional coactivator SRC-3 by the 20S proteasome [28]. PSME3 may similarly promote the degradation of PPM1D by facilitating its recruitment to the 20S proteasome. The C-terminus of PPM1D contains an unstructured region that can be directly recognized by the 20S proteasome, as well as binding sites for PSMD14 and PSME3, which promote degradation. This notion is consistent with the observed stabilization of the C-terminal truncation mutant of PPM1D in HCT116 and U2OS cells. Further analysis of the IDR in the C-terminal 35 amino acid residues is essential to identify more specific degrons that regulate PPM1D degradation.

Our findings also suggest that PPM1D is typically maintained at a constitutively low level through rapid, ubiquitin-independent proteasomal degradation, a mechanism that enables cells to mount an immediate response to diverse stimuli. For instance, upon DNA damage, the low basal abundance of PPM1D permits swift activation of the p53 pathway, thereby facilitating cell cycle arrest and the induction of DNA repair programs. By contrast, ubiquitin-dependent degradation plays a secondary role under basal conditions but is well suited for contexts requiring more gradual and precise regulation, such as the stepwise adjustment of PPM1D levels during response termination. When DNA damage is assessed to be repairable, suppression of the ubiquitin-independent proteasomal pathway stabilizes PPM1D, leading to a progressive attenuation of p53 activity and allowing cell cycle re-entry. Conversely, when the damage is severe and irreparable, activation of the ubiquitin-dependent proteasomal pathway maintains PPM1D at low levels, thereby sustaining p53 activity and promoting irreversible cell fate decisions, such as apoptosis. Thus, the coexistence of these two degradation mechanisms may allow cells to modulate the rate and mode of PPM1D turnover according to the nature and severity of stimuli, as well as the repairability of damage, thereby fine-tuning p53 activity to ensure appropriate cell fate determination.

Recently, targeted protein degradation has attracted significant attention in the field of drug discovery as a promising approach to eliminating disease-associated proteins. One of the most well-established targeted protein degradation technologies, PROTAC (PROteolysis TArgeting Chimera), induces the degradation of a protein of interest by bringing it into proximity with a ubiquitin ligase, leading to ubiquitin-dependent proteasomal degradation [35]. However, the number of ubiquitin ligases currently available for PROTAC applications is limited, and extensive research is being conducted to expand its applicability. In this context, the direct recruitment of proteins of interest to the proteasome using small molecules has been explored as an alternative targeted protein degradation strategy. A ligand for PSMD2, a subunit of the 19S proteasome, has been identified, and a bifunctional molecule combining this ligand with a BRD4-binding ligand via a linker has been reported to direct BRD4 to the UbInPD pathway [36]. Additionally, a bifunctional molecule comprising a HaloTag ligand and a bromodomain ligand was used to induce the UbInPD of BRD2 through the fusion of HaloTag with the proteasome subunit RPN13 [37]. Although this molecule was also capable of binding to BRD4, a paralog of BRD2, it failed to induce its degradation, suggesting that specific conditions are required for effective UbInPD. One crucial factor may be the spatial orientation of the protein of interest relative to the proteasome upon recruitment. Therefore, understanding the relationship between endogenous UbInPD substrates and their respective proteasomal receptors will be essential for designing effective small-molecule degraders. We demonstrated that PSMD14 and PSME3 facilitate the degradation of PPM1D. Given their roles in promoting proteasomal degradation, these proteasome subunits may serve as potential targets for future drug development aimed at inducing UbInPD.

Previous research on PPM1D inhibitors in mantle cell lymphoma suggested that p53 activation and apoptosis induction were the primary mechanisms for lymphoma suppression [38]. Furthermore, Inhibition of PPM1D sensitizes breast cancer cells to genotoxic stress, leading to cell cycle delay, G2 checkpoint activation, and p53 pathway activation, thereby promoting apoptosis [39]. Based on these findings, PPM1D inhibitors have been proposed as a potential therapeutic agent for malignant tumors [40]. In this study, we also tested the hypothesis that the accumulation of PPM1D due to proteasome inhibition contributes to tumor cell survival, similar to the increased malignancy observed with C-terminal deletion mutants of PPM1D. In A549, U87MG, HCT116, and U2OS cells, bortezomib treatment increased PPM1D protein levels, and the combination of bortezomib with the PPM1D inhibitor GSK2830371 synergistically suppressed cell proliferation. Notably, co-treatment with the PPM1D inhibitor GSK2830371 synergistically enhanced the antiproliferative effect of bortezomib in all tested cell lines, as demonstrated by Chou–Talalay analysis. The synergistic effect was more pronounced at higher fractional effect level, indicating that simultaneous inhibition of the proteasome and PPM1D is particularly effective under stronger proliferative pressure. These findings suggest that co-inhibition of the proteasome and PPM1D effectively suppresses tumor progression, at least in cells harboring wild-type p53. However, the observation that a synergistic effect was also seen in some non-tumorigenic cell lines raises the possibility that this strategy may affect not only malignant but also certain normal cells. This highlights a potential limitation regarding therapeutic selectivity and underscores the need for further investigation into the safety and specificity of this combination. Despite these concerns, our findings provide a compelling rationale for continued exploration of proteasome and PPM1D co-inhibition as a novel approach in cancer therapy.

## Conclusions

This study revealed that one of the mechanisms underlying PPM1D degradation is UbInPD mediated via its C-terminal 35 amino acid residues. Specifically, we demonstrated that PPM1D is directly degraded by the 20S proteasome, which targets its C-terminal IDR, and that PSMD14 and PSME3 may function as activators in this process. Furthermore, we demonstrated that the combination of a proteasome inhibitor and a PPM1D inhibitor synergistically enhances the antitumour effect, suggesting a potential therapeutic strategy for malignancies.

## Supplementary Information

Supplementary material 1 (docx 2818 KB)

## Data Availability

The mass spectrometric datasets were deposited in ProteomeXchange (RRID:SCR_004055) under the accession number PXD060414 via the jPOST repository. Other datasets supporting the findings of this study are available from the corresponding author on reasonable request.

## Abbreviations

UbInPD: Ubiquitin-independent proteasomal degradation
CI: Combination index
Fa: Fraction affected
SD: Standard deviation
IDR: Intrinsically disordered regions
NQO1: NAD(P)H:quinone oxidoreductase-1
