# Supplementary material for: PPM1D is directly degraded by proteasomes in a ubiquitination-independent manner through its carboxyl-terminal region
Source: J Biomed Sci. 2025 Sep 11;32:88. doi: 10.1186/s12929-025-01185-z (PMC12424213; doi:10.1186/s12929-025-01185-z)
Supplement: Supplementary file 1 — Supplementary material 1 (docx 2818 KB) [file 12929_2025_1185_MOESM1_ESM.docx]

Supplementary Fig. S1


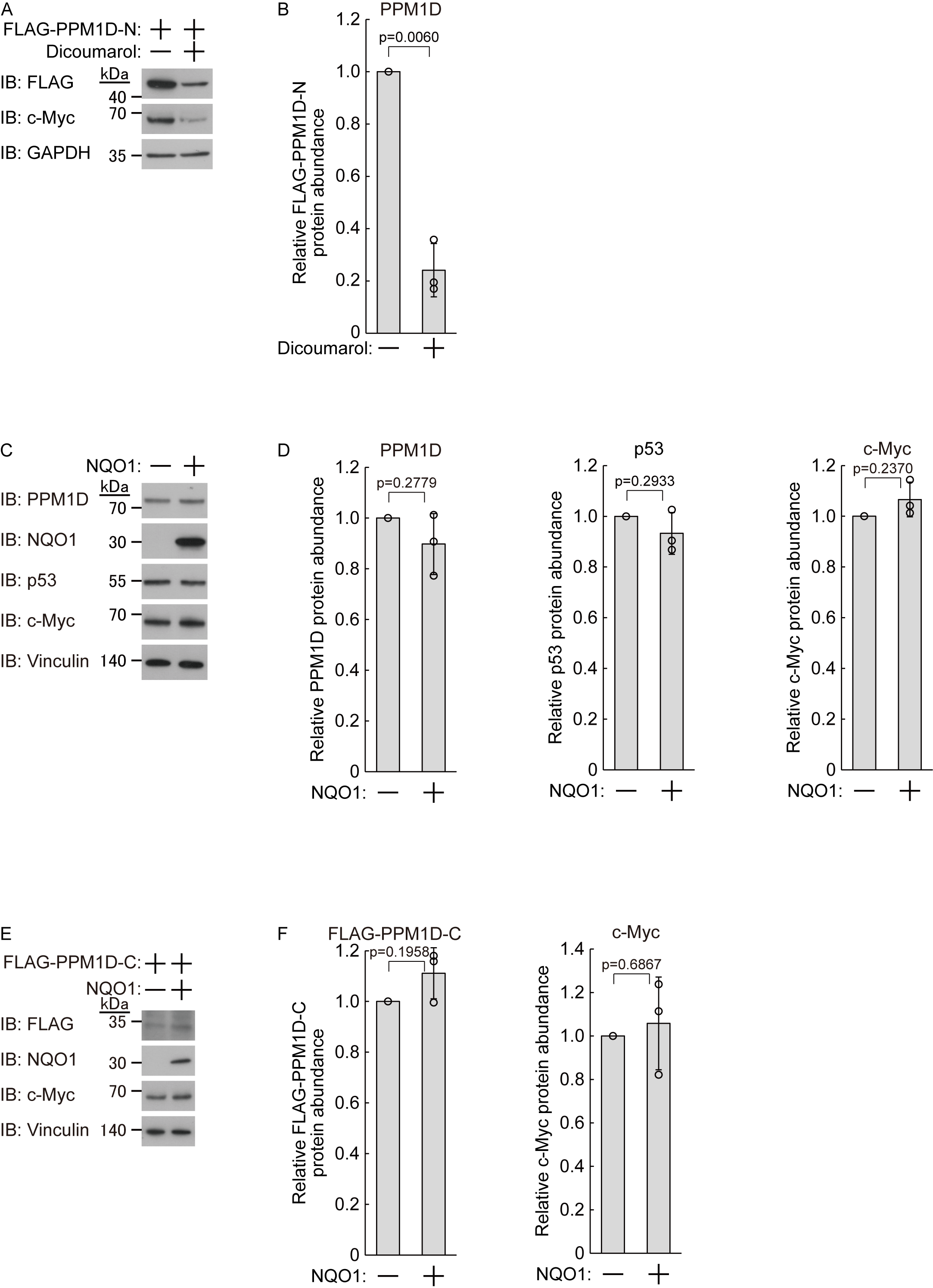


**Supplementary Fig. S1: Analysis of the effects of dicoumarol and NQO1 expression on PPM1D protein levels. A, B** HEK293T cells stably expressing FLAG-tagged PPM1D-N were treated for 3 hours with dicoumarol (300 μM) (n = 3). Representative immunoblots (**A**) and quantification (**B**) of PPM1D protein levels are shown. Protein levels were normalized to GAPDH. **C, D** PPM1D protein levels were analyzed in HEK293T cells stably expressing NQO1 and in corresponding control cells. Representative immunoblots (**C**) and quantification (**D**) are shown. Protein levels were normalized to vinculin. **E, F** HEK293T cells stably co-expressing FLAG-tagged PPM1D-C and NQO1, or expressing FLAG-tagged PPM1D-C alone, were analyzed. Representative immunoblots (**E**) and quantification (**F**) of PPM1D protein levels are shown. Protein levels were normalized to vinculin.

Supplementary Fig. S2


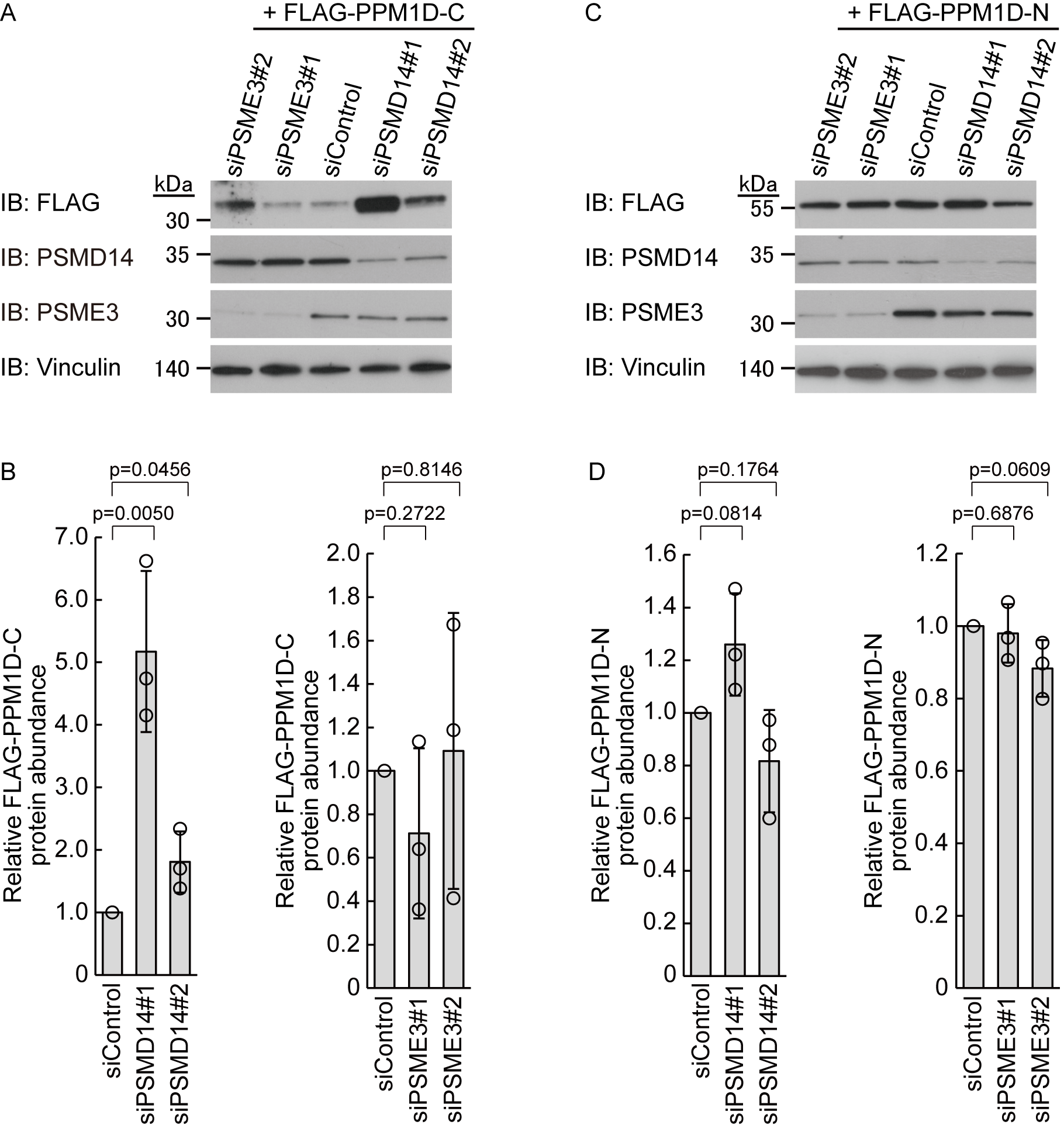


**Supplementary Fig. S2: Effects of PSME3 and PSMD14 knockdown on PPM1D-N and PPM1D-C mutant protein levels.** **A–D** HEK293T cells stably expressing FLAG-tagged PPM1D-N (**A, B**) or PPM1D-C (**C, D**) mutants were transfected with siRNAs targeting PSME3 (n = 3) and PSMD14 (n = 3). After 48 hours, cells were harvested and subjected to immunoblot analysis using antibodies against FLAG, PSME3, PSMD14, and vinculin. Representative immunoblots (**A, C**) and quantification (**B, D**) of PPM1D protein levels are shown. Protein levels were normalized to vinculin. Data represent the mean ± SD.

Supplementary Fig. S3


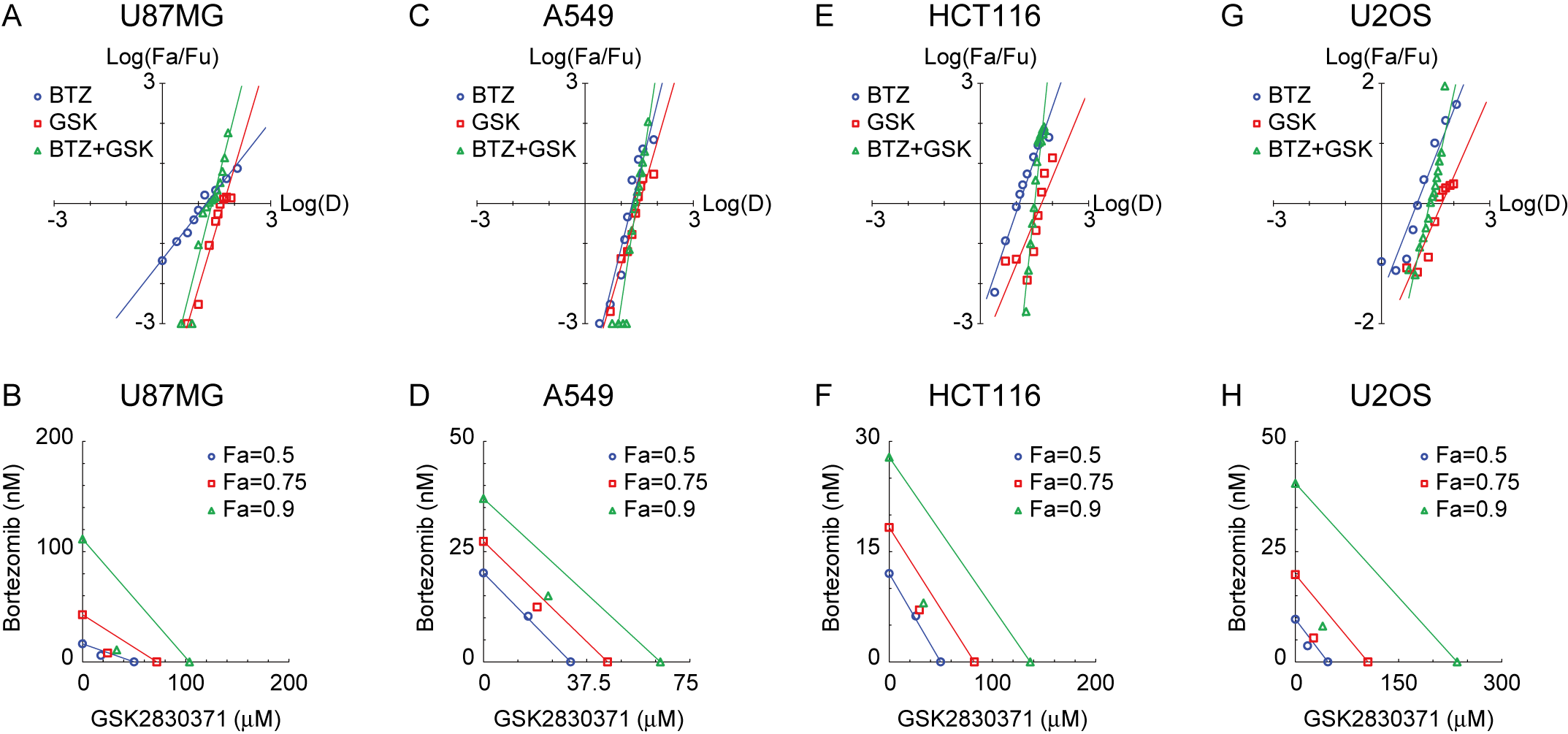


**Supplementary Fig. S3: Median-effect plots and isobolograms demonstrating the combination effects of bortezomib and GSK2830371 in p53 wild-type cancer cell lines.** Median-effect plots (**A, C, E, G**) and corresponding isobolograms (**B, D, F, H**) were generated using the Chou–Talalay method for U87MG (**A–B**), A549 (**C–D**), HCT116 **(E–F**), and U2OS (**G–H**) cells treated with various concentrations of bortezomib and GSK2830371 at a fixed molar ratio based on their IC_50_ values. The median-effect plots assess the conformity of dose–response data to the mass-action law. Fa and Fu represent the fractional effect and the fraction unaffected (Fu = 1 − Fa), respectively. D denotes the dose of drug required to achieve the given effect. The isobolograms depict the combination doses at different Fa levels, comparing them to the theoretical additive line. Data points below the line indicate synergy between the two drugs.

Supplementary Fig. S4


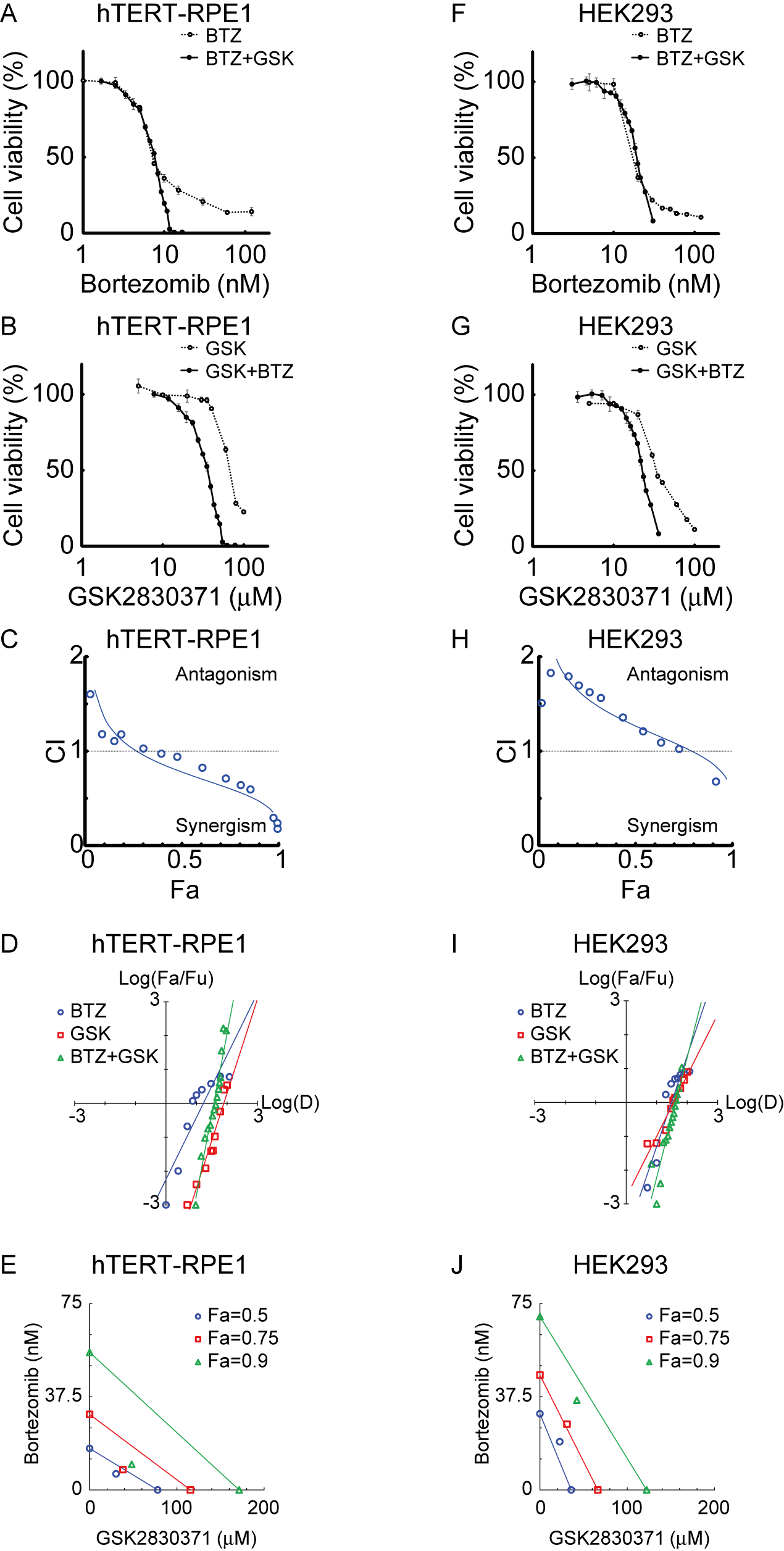


**Supplementary Fig. S4: Evaluation of the combinatorial effects of bortezomib and GSK2830371 in immortalized non-cancerous cell lines.** HEK293 (**A–E**) cells and hTERT-RPE1 (**F–J**) cells were treated with bortezomib and GSK2830371 at various concentrations using a fixed molar ratio based on each drug’s IC_50_. **A, F** Dose–response curves comparing bortezomib alone and in combination with GSK2830371. **B, G** Dose–response curves comparing GSK2830371 alone and in combination with bortezomib. **C, H** Combination Index (CI) plots generated using the Chou–Talalay method, showing the relationship between the fraction affected (Fa, x-axis) and CI (y-axis). CI values < 1 indicate synergistic interaction between the two drugs. **D, I** Median-effect plots used to evaluate the fit of the dose–response data to the median-effect principle. Fu represents the fraction unaffected (Fu = 1 − Fa), and D denotes the dose of drug required to achieve the given effect. **E, J** Isobolograms demonstrating the interaction between the two drugs at multiple effect levels. Synergy is indicated when CI < 1 or when data points fall below the line of additivity in the isobologram.

Supplementary Fig. S5


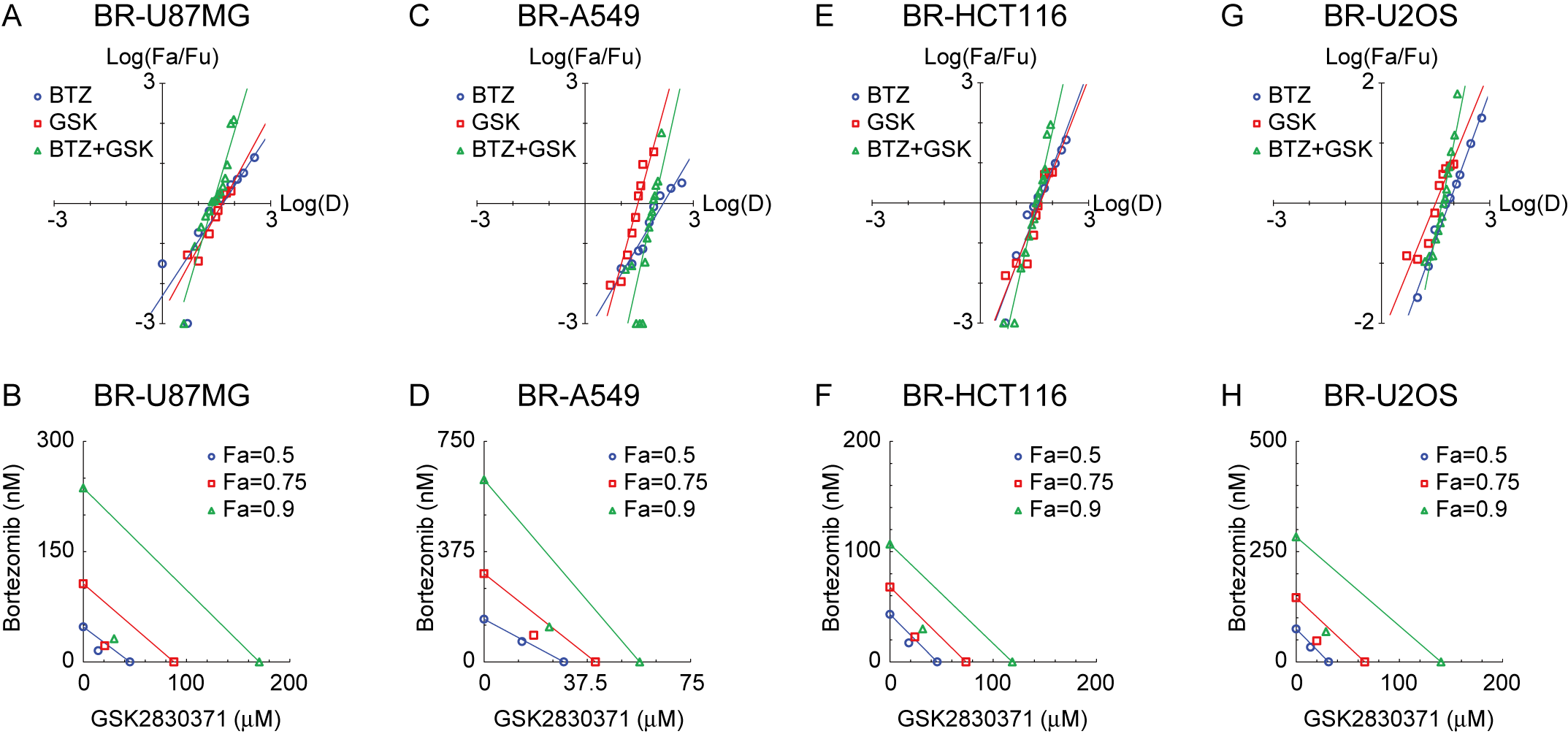


**Supplementary Fig. S5: Median-effect plots and isobolograms for bortezomib-resistant cancer cell lines treated with bortezomib and GSK2830371.** Median-effect plots (**A, C, E, G**) and corresponding isobolograms (**B, D, F, H**) were generated using the Chou–Talalay method for bortezomib-resistant (BR) variants of U87MG (**A, B**), A549 (**C, D**), HCT116 (**E, F**), and U2OS (**G, H**) cells. Cells were treated with bortezomib and GSK2830371 at various concentrations using a fixed molar ratio based on the IC_50_ values of the respective resistant lines. The median-effect plots assess the dose–effect relationship and the conformity of the data to the mass-action law. Fa and Fu represent the fractional effect and the fraction unaffected (Fu = 1 − Fa), respectively. D denotes the dose of drug required to achieve the given effect. The isobolograms illustrate the combination doses at different Fa levels and their deviation from the line of additivity, with points below the line indicating synergy between the drugs.
